# Transcription elongation mechanisms of RNA polymerases I, II, and III and their therapeutic implications

**DOI:** 10.1016/j.jbc.2024.105737

**Published:** 2024-02-08

**Authors:** Ruth Q. Jacobs, David A. Schneider

**Affiliations:** Department of Biochemistry and Molecular Genetics, University of Alabama at Birmingham, Birmingham, Alabama, USA

**Keywords:** RNA polymerase I, RNA polymerase II, RNA polymerase III, transcription elongation, kinetics, BMH-21

## Abstract

Transcription is a tightly regulated, complex, and essential cellular process in all living organisms. Transcription is comprised of three steps, transcription initiation, elongation, and termination. The distinct transcription initiation and termination mechanisms of eukaryotic RNA polymerases I, II, and III (Pols I, II, and III) have long been appreciated. Recent methodological advances have empowered high-resolution investigations of the Pols’ transcription elongation mechanisms. Here, we review the kinetic similarities and differences in the individual steps of Pol I-, II-, and III-catalyzed transcription elongation, including NTP binding, bond formation, pyrophosphate release, and translocation. This review serves as an important summation of *Saccharomyces cerevisiae* (yeast) Pol I, II, and III kinetic investigations which reveal that transcription elongation by the Pols is governed by distinct mechanisms. Further, these studies illustrate how basic, biochemical investigations of the Pols can empower the development of chemotherapeutic compounds.

### DNA-dependent RNA polymerases in the three domains of life

All cells rely on DNA-dependent RNA polymerases (RNAPs) for gene expression. They are responsible for transcribing DNA to synthesize RNA. Resultant RNA is coding or non-coding. Coding RNAs are messenger RNAs (mRNA) that are translated by ribosomes to synthesize polypeptide chains. The vast majority of the cellular RNA is non-coding. Over 95% of cellular RNAs are ribosomal RNA (rRNA) and transfer RNA (tRNA). Outside of rRNA and tRNA, the remaining non-coding transcripts are generally small (<1 kb) and regulate gene expression by influencing chromatin remodeling; enhancement or suppression of transcription; mRNA turnover, stability, and decay; and promotion or suppression of translation (1, 2, 3).

Prokaryotes express a single RNAP that is solely responsible for synthesizing all the RNA required by the cell. In contrast, eukaryotes express at least three nuclear RNA polymerases (Pols I, II, and III) (4). It is theorized that the RNAPs present in three domains of life, Archaea (5, 6, 7), Bacteria (8, 9, 10, 11, 12), and Eukarya (13, 14, 15, 16, 17), share a common ancestral RNAP. This is structurally supported by five well-conserved subunits and the shared double-ψ β-barrel domains present in the active center. The active center is formed by the two largest RNAP subunits in Bacteria, Archaea, and Eukarya (18, 19, 20). In this review, we focus on the functional divergence of the *Saccharomyces cerevisiae* (yeast) Pols.

Extensive structural comparisons of the Pols have been detailed by many (21, 22, 23, 24, 25). Briefly, Pol I is composed of 14 subunits (16), Pol II is composed of 12 subunits (13, 14), and Pol III is the largest Pol with 17 subunits (17). Unlike the bacterial RNAP, the homologous five subunit core in eukaryotes is insufficient to form a transcriptionally active Pol (26). Pols I, II, and III require four additional subunits that are shared, and a fifth subunit of similar structure and function. Therefore, the Pols share a structurally and functionally conserved 10-subunit catalytic core. Outside of the 10-subunit eukaryotic core, there are four subunits unique to Pol I, two to Pol II, and seven to Pol III.

These structural differences between the Pols likely facilitate the transcription of their unique genetic targets. Pol I is the most specialized Pol in the eukaryotic system because it has one target gene, the 35 S ribosomal DNA (rDNA) in yeast. The 35 S rDNA is organized in ∼150 tandem repeats on chromosome XII (27, 28). Each 9.1 kb repeat includes the 18 S, 5.8 S, and 25 S rRNAs (29). The rDNA repeats are found in either a nucleosome-bound or a nucleosome-free organization, which correspond to the transcriptionally inactive or active repeats, respectively (30, 31). Transcription of the rDNA occurs in a specialized compartment of the nucleus, the nucleolus.

In the nucleus, Pol II synthesizes mRNA and diverse classes of non-coding RNAs, including long non-coding RNA, small nucleolar RNA, small nuclear RNA, and micro RNA. Pol II is responsible for the largest number of genes (32) and requires the most transcription-associated proteins to facilitate the synthesis of RNA products from chromatinized templates (33, 34, 35, 36, 37), which have been extensively reviewed (22, 38, 39, 40, 41).

Pol III is more specialized than Pol II but less than Pol I. Pol III is responsible for transcribing approximately 300 genes in yeast (42), half of which are nucleosome-bound (43). Pol III only synthesizes short, non-coding RNAs including the 5 S rRNA, tRNA, U6 spliceosomal RNA, and 7SL RNA of RNase P (42, 44, 45). Through Pol-specific transcription factors (TFs), eukaryotes can modulate the activity of one Pol, without impacting the other two, which ultimately offers dynamic regulation of specific gene classes required by higher eukaryotes.

### Pols possess distinct transcription initiation and termination mechanisms

All Pols share the same basic transcription cycle – transcription initiation, transcription elongation, and transcription termination. For decades, it has been established that Pols I, II, and III have distinct transcription initiation mechanisms (25). Pol I transcription initiation *in vivo* is a stepwise process (46) that requires the binding of upstream activating factor (47, 48, 49), TATA-binding protein (TBP) (50, 51, 52, 53), core factor (CF), Rrn3, and Pol I at the rDNA promoter. Unlike the single promoter for Pol I transcription, Pol II must initiate transcription at a variety of promoters and these core promoter regions are highly diverse in sequence (54). In general, Pol II transcription initiation begins in a closed-complex state and requires the assistance of TFIIA, TFIIB, TFIID (contains TBP and TBP-associated factors), TFIIE, TFIIF, and TFIIH (39, 55). Unlike Pols I and III, Pol II requires ATP-hydrolysis and TFIIH’s helicase activity to unwind the DNA and form an open-complex (56, 57). Finally, Pol III initiates transcription at three different gene promoters, two have gene internal elements: Type I (5 S rDNA) and Type II (tDNA); whereas Type III (U6 gene) promoters have regulatory elements upstream of the transcriptional start site (58, 59). Type I promoters require TFIIIA (60), TFIIIC, and TFIIIB, Type II promoters require TFIIIC and TFIIIB, and Type III promoters are bound by snRNA activating protein complex and TFIIIB. The commonality among the Pol III promoters is the binding of TFIIIB, which is composed of three subunits: TBP, Brf1, and Bdp1. TFIIIB is required to recruit Pol III to the DNA. The Pols’ distinct transcription initiation processes and Pol-specific TFs allow the eukaryotic cell to regulate the expression of specific genes without influencing the genes transcribed by another Pol.

It is also known that Pol I, II, and III transcription termination mechanisms are different. Pol I terminates at either the +93 or +250 termination site downstream of the 3′ end of the rDNA 25 S gene region (29, 61). These sites are not used equally, rather, 90% of Pol I transcripts are terminated at the +93 site (with the help of Reb1 (62, 63)), while the +250 site acts as a fail-safe (61). Both Reb1 and a T-rich stretch of DNA pause and destabilize Pol I transcription elongation complexes (ECs), which causes Pol I dissociation from the rDNA and the release of the 35 S rRNA (62). Pol II transcription termination is a complex process that is dependent upon the transcript synthesized (64). For protein-coding transcripts, the poly(A) signal recruits the binding of 3′ end processing factors to release the RNA from Pol II ECs (65, 66). An additional protein, Rat1, is required to cause Pol II termination. The exoribonuclease activity of Rat1 allows it to degrade the RNA protruding from the Pol II EC from the 5′ end. Ultimately, collision between Rat1 and Pol II triggers Pol II’s disengagement from the DNA. In contrast, poly(A)-independent termination requires the association of two RNA binding proteins, Nrd1 and Nab3, and a helicase, Sen1 (67, 68). Pol III transcription termination can be achieved in a factor-independent manner. Transcription of all Pol III target genes are terminated by a universal oligo(dT) sequence. A stretch of T’s in the non-template DNA, the required length of which varies by species, cause a weak RNA:DNA hybrid which destabilizes Pol III ECs (69). It is evident that over the course of evolution, each Pol achieved EC disengagement and release of the template DNA *via* different mechanisms.

### Individual steps of transcription elongation

Despite the differences between Pol I, II, and III transcription that we have summarized above, it was previously expected that their transcription elongation mechanisms would be very similar. This was a reasonable hypothesis because the Pols have conserved structure and sequence around the active center (24), the Pols have been shown to exhibit similar elongation rates *in vivo* (Pol I, ∼60 nt s^−1^ (70); Pol II, ∼33 nt s^−1^ (71); Pol III, ∼61 nt s^−1^ (72)), and the required chemical steps of nucleotide incorporation are the same for all Pols.

We know that transcription elongation is a repetition of at least four essential steps – NTP binding, bond formation, pyrophosphate (PPi) release, and translocation (Fig. 1). NTP binding by Pols I (73), II (74, 75), and III (76) has been described as a diffusion-controlled, rapid equilibrium process. Interestingly, Pols I (73), II (75), and III (76) share a similar binding affinity for ATP while the binding affinity for all NTPs has only been investigated for Pol II.

**Figure 1:**
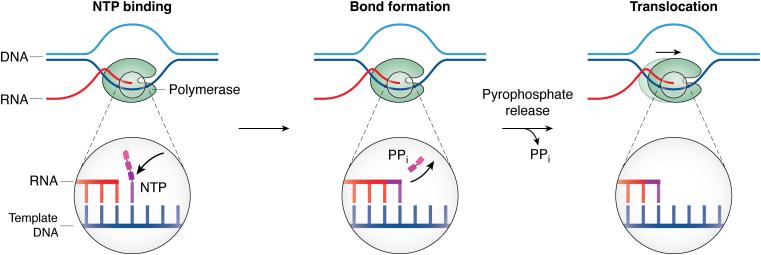
**Transcription elongation requires the repetition of NTP binding, bond formation, pyrophosphate release, and translocation**.

Several research groups have examined bond formation by Pols I, II, and III using different *in vitro* techniques. Here, we report calculated rates and rate constants for each Pol. Although the rate of bond formation varies within the Pol I, II, and III literature (73, 75, 76, 77, 78), we can generally conclude that Pols I and III are substantially faster than Pol II. Unfortunately, no study to date has directly monitored the kinetic mechanism of PPi release by the Pols. As a result of this knowledge gap, some groups have reported a single kinetic measurement that sums both the rate of bond formation and PPi release (77, 78).

Finally, translocation kinetics have been reported for Pol II only (77, 78), while translocation by Pols I and III remain to be tested. Various research groups have added additional kinetic parameters to fit their transcription elongation data, but our focus will be on the essential steps that have been directly experimentally investigated, NTP binding, bond formation, and translocation.

Lastly, we discuss the implications of the Pols’ kinetic differences in the modern cancer therapeutic field. In recent decades, Pol I has emerged as a viable anti-cancer target. Specifically, we focus on a small-molecule inhibitor, BMH-21, and how it may target the unique kinetic properties of Pol I to achieve its chemotherapeutic effect in a Pol-specific manner. We exclusively review studies that examine the mechanisms of transcription elongation by the yeast Pols, because yeast is as an excellent and reliable eukaryotic model.

## Pol I transcription elongation kinetics

### Pol I transcription elongation rate estimations *in vivo* and *in vitro*

Electron microscopy (EM) analysis of rDNA repeats *in vivo* empowered the estimation of an elongation rate of ∼60 nt s^−1^ for Pol I (70) (Table 1). To reach this value, the Beyer group counted the number of actively engaged Pol I ECs on the 35 S rDNA and determined how fast Pol I ECs must synthesize 35 S rRNA transcripts to support the production of 2000 ribosomes per minute (estimated rate of ribosome synthesis in actively growing yeast (79)). It is important to highlight assumptions made by the researchers to arrive at a rate of ∼60 nt s^−1^; they assumed that each engaged Pol EC would yield a 35 S rRNA transcript and that the 35 S rRNA transcript would be productively incorporated into a ribosome. First, not all transcription elongation events are processive, meaning, not all elongating Pols yield a full-length product. Pols can undergo pausing, arrest, backtracking, or destabilization prior to full transcript synthesis which would result in the degradation of those incomplete RNAs. Secondly, in absence of knowing the 35 S rRNA synthesis and decay rate, we do not know if each 35 S rRNA transcript is used for ribosome biogenesis. Taken together, it is possible that the EM estimation of Pol I’s elongation rate, ∼60 nt s^−1^ (70), is an underestimation.

**Table 1:** Pol I transcription elongation kinetic parameters

Pol I			
Parameter	Value	Method	Source
General transcription elongation estimations			
Elongation rate *in vivo*	∼61 nt s^−1^	EM analysis of 35 S rDNA	(70)
Elongation rate *in vitro*	∼58 nt s^−1^	Modified rDNA template (∼800 bp)	(81)
	∼67 nt s^−1^		(82)
	∼53 nt s^−1^	Synthetic DNA template encoding four AMP and five GMP incorporations	(76, 87)
Individual steps of nucleotide addition			
NTP binding	K_1/2_ = ∼140 μM NTP	Modified rDNA template (∼800 bp)	(82)
	K_1/2_ = ∼170 μM ATP	Synthetic DNA template encoding one AMP incorporation	(73)
Bond formation	∼180 s^−1^	Synthetic DNA template encoding one AMP incorporation	(73)
Translocation		undetermined	

*In vivo* experiments were carried out at 30 °C and *in vitro* experiments were executed at (23–25) °C when noted by the authors.

Pol I elongation rates have also been measured *in vitro* with a fully reconstituted transcription assay (80). The Finzi and Schneider research groups measured similar maximal Pol I elongation rates, 58 nt s^−1^ (81) and 67 nt s^−1^ (82), respectively (Table 1). Additionally, Schneider and colleagues titrated [NTPs] and determined a K_1/2_ of 142 μM (82). Interestingly, these *in vitro* values are in good agreement with the EM estimation of ∼60 nt s^−1^. Throughout this review, we will compare kinetic parameters measured *in vivo* and *in vitro*. However, it is important to emphasize that *in vitro* and *in vivo* conditions differ significantly.

### Pol I single nucleotide addition mechanism

To further understand the elementary kinetic parameters of nucleotide addition by Pol I, the Schneider and Lucius research groups designed a promoter-independent *in vitro* transcription assay to monitor the extension of a 10-mer RNA to an 11-mer RNA with the incorporation of a single AMP (73, 83). They calculated kinetic parameters governing the appearance of the 11-mer by globally fitting the data to a minimal AMP incorporation reaction scheme. They determined that Pol I binds ATP at a K_1/2_ of ∼170 μM and bond formation occurred at ∼180 s^−1^ (Table 1). It is important to note that they did not monitor translocation, an essential step of nucleotide addition, because experimentally, Pol I ECs were halted after AMP incorporation by limiting for the next encoded NTP.

To determine if bond formation is the rate-limiting step of AMP addition, a slowly hydrolysable ATP analog, Sp-NTP-α-S, can be used to slow down bond formation. If bond formation is rate-limiting, a > 10-fold decrease in the rate constant is expected (84, 85, 86). It has been shown that replacing ATP with Sp-ATP-α-S led to a ∼ 35-fold decrease in Pol I’s rate of bond formation (82). This finding suggests that Pol I AMP addition is rate-limited by bond formation. Is the mechanism of Pol I AMP incorporation indicative of all NMP incorporations by Pol I? In order to understand the impact of NTP sequence on Pol I incorporation kinetics, future studies of single GMP, CMP, and UMP addition are required.

### Pol I multi-nucleotide addition reveals sequence effects

While single nucleotide experiments empower the study of the core activity of Pol I, we do not know if processive elongation is a simple repetition of the single nucleotide addition reaction or if there are intermediate steps. To address this question, the Lucius and Schneider research groups monitored the incorporation of nine nucleotides (four AMP incorporations, five GMP incorporations) (87). Importantly, this experimental setup is inherently sensitive to translocation because multiple nucleotides are incorporated instead of one. If the mechanism of Pol I single AMP incorporation can be extrapolated to describe multi-nucleotide addition, one would expect the single AMP bond formation rate constant, ∼180 s^−1^ (73), to be rate-limiting for multi-nucleotide addition as well. We find that the hypothesis is unsupported; the average multi-nucleotide addition rate constant was substantially slower, ∼53 nt s^−1^ (Table 1). Interestingly, the appearance of each of the nine RNAs was governed by a different rate constant (87). This raises the question of, what governs the rate of the addition?

If NTP identity alone determined the rate of a nucleotide addition event, one would expect the four AMP incorporations to occur at the same rate and the five GMP incorporations to occur at the same rate. Instead, Ingram *et al*. found that Pol I incorporates AMPs and GMPs at dissimilar rate constants (87). Pol I incorporates multiple nucleotides in a heterogenous pattern, with an 8.5-fold difference between the fastest and slowest rate constant. What is driving the variability in Pol I nucleotide addition rate constants? It is likely a cumulative effect of the identity of the incorporated NMP and local DNA context. Perhaps the sequence upstream and downstream of the EC alters the rate of any given NMP incorporation because of different Pol-nascent RNA interactions. Furthermore, it is also possible that secondary nascent RNA structures forming behind the Pol may induce faster or slower additions. Ultimately, there are several variables that may explain the disparity in measured single and multi-nucleotide addition rates that require further investigation.

In conclusion, these Pol I studies reveal that Pol I single AMP incorporation is rate-limited by bond formation and that Pol I multi-nucleotide addition is described by different rate constants at each position, likely due to DNA sequence effects. Key questions that remain unanswered include, what is the mechanism of Pol I translocation? How do different NTPs (GTP, CTP, UTP) influence the rate-limiting step of nucleotide addition? To what degree does the identity of the encoded NTP, local DNA context, and nascent RNA interactions/structures influence Pol I elongation kinetics?

## Pol II transcription elongation kinetics

### Pol II transcription elongation rate estimations *in vivo* and *in vitro*

Pol II transcription elongation has been subjected to more *in vivo* and *in vitro* investigations than Pols I or III. The Struhl research group measured the transcription elongation rate of Pol II on a non-essential gene controlled by the *GAL1* promoter (71). They transferred the yeast from galactose media to glucose-containing media and monitored the “last wave” of Pol II ECs. They determined that Pol II elongates at a rate of ∼33 nt s^−1^ (Table 2). Slower elongation rates have been reported *in vitro*; Bustamante and colleagues employed single-molecule optical tweezers to determine an elongation rate of 12 nt s^−1^ on a 9.8 kb DNA template (88) (Table 2). Interestingly, the Schneider group found a similar *in vitro* rate on a significantly shorter template (76, 89): Pol II extended a 10-mer RNA to a 19-mer RNA at an average elongation rate of 9 nt s^−1^ (Table 2). Taken together, these investigations reveal that Pol II is a slower enzyme, more similar to the elongation rate of *Escherichia coli* RNAP (90) than Pol I.

**Table 2:** Pol II transcription elongation kinetic parameters

Pol II			
Parameter	Value	Method	Source
General transcription elongation estimations			
Elongation rate *in vivo*	∼33 nt s^−1^	*YLR454* under GAL1 promoter	(71)
Elongation rate *in vitro*	∼12 nt s^−1^	Optical tweezers, ∼9.8 kb template	(88)
	∼9 nt s^−1^	Synthetic DNA template encoding four AMP and five GMP incorporations	(76, 89)
Individual steps of nucleotide addition			
NTP binding	K_d_ = ∼140 μM NTP	Optical tweezers, ∼4 kb template	(77)
	K_M_ = ∼40 μM NTP	Optical tweezers, ∼3 kb template	(78)
	K_1/2_ = ∼110 μM ATP	Synthetic DNA template encoding one AMP incorporation	(75)
Bond formation	∼34 s^−1^	Optical tweezers, ∼4 kb template	(77)
	∼35 s^−1^	Optical tweezers, ∼3 kb template	(78)
	∼75 s^−1^	Synthetic DNA template encoding one AMP incorporation	(75)
	∼61 s^−1^	Synthetic DNA template encoding one CMP incorporation	(92)
	∼75 s^−1^	Synthetic DNA template encoding one CMP incorporation	(91)
Translocation	[pre-]/[post-] = 0.2	Optical tweezers, ∼4 kb template	(77)
	Forward = ∼88 s^−1^ Reverse = ∼680 s^−1^	Optical tweezers, ∼3 kb template	(78)

*In vivo* experiments were carried out at 30 °C and *in vitro* experiments were executed at (23–25) °C when noted by the authors.

### Pol II nucleotide addition mechanisms *in vitro*

While many *in vitro* studies have offered models of Pol II transcription elongation, here, we focus on the few studies that have determined kinetic parameters that describe the reaction. The Block lab employed an optical-trapping assay to monitor individual elongating Pol II complexes and determined a best-fit model (77). They found that Pol II can bind NTP in a pre- or post-translocated state, governed by a K_d_ of ∼140 μM NTP, and both bond formation and PPi release were described by a single rate constant, ∼34 s^−1^ (Table 2). Additionally, they reported a translocation equilibrium constant that suggested a bias toward Pol II complexes in the post-translocated state (Table 2). In contrast, the Kashlev group used DNA footprinting and determined that majority of Pol II ECs resided in the pre-translocated state (91). Using a similar optical-trapping assay to the Block group, the Bustamante group added the variable of a nucleosomal barrier and similarly found a Pol II EC bias toward the pre-translocated state (78) (Table 2). Further, Bustamante and colleagues determined that Pol II binding of NTP was governed by a substantially smaller Michaelis constant, they calculated a K_M_ of ∼40 μM NTP (Table 2). Despite these differences, they determined that bond formation and PPi release occurred at ∼35 s^−1^ (Table 2). Not only are the catalysis rate constants calculated by the Block and Bustamante groups in good agreement, but their values are also consistent with the *in vivo* estimation made by the Struhl lab (71). Taken together, the agreement in Pol II catalysis rate between the *in vivo* and *in vitro* studies support the notion that Pol II is slower that Pols I and III.

### Pol II bond formation and translocation may be partially rate-limiting

The Bustamante group found that rates of forward translocation and bond formation are within an order of magnitude (Table 2). This suggests that Pol II is partially rate-limited by two steps, translocation and bond formation. This finding is supported by a kinetic model proposed by the Lucius and Schneider labs (75). They found that after Pol II ECs bind ATP, described by a K_d_ of ∼110 μM, two steps occur, bond formation, at ∼75 s^−1^, and an unknown slow step, at ∼3 s^−1^ (Table 2). Interestingly, their calculated AMP bond formation rate constant, ∼75 s^−1^, is in good agreement with the apparent maximal incorporation rates of CMP calculated by the Burton and Kashlev groups, ∼61 s^−1^ (92) and ∼75 s^−1^ (91). This suggests Pol II may incorporate different NMPs at a similar rate, contrasting what was observed for Pol I by Ingram *et al*. (87).

The Lucius and Schneider labs observed a ∼25-fold reduction in the rate constant governing bond formation (75), in the presence of Sp-ATP-α-S compared to ATP. This suggests that bond formation is one of the partially rate-limiting steps for single nucleotide addition by Pol II. The identity of the slow step, ∼3 s^−1^, was not discerned. However, it is worth noting that the rate constants governing these two steps are in the same order of magnitude, consistent with the findings of Bustamante and colleagues. It is possible that the unknown conformational change observed by the Lucius and Schneider groups corresponds to translocation, but the substantial difference in the rate constant describing the step, ∼3 s^−1^, and forward translocation calculated by Bustamante and colleagues, ∼88 s^−1^ (78), renders this possibility unlikely (Table 2).

If the slow step is not translocation itself, what is that step describing? From molecular-dynamics simulations by the Burton lab (93) and the Huang and Wang research groups (94), we know that catalysis by Pol II requires a closed trigger loop, that PPi release is coupled with trigger loop opening, and that translocation primarily occurred with an open trigger loop. Therefore, it possible the slow, unknown conformational change observed by Lucius and Schneider was a shift to or the persistence of a closed trigger loop conformation. Potentially in support of this hypothesis, the Lahiri and Pata labs found that the bacterial DNA polymerase can sense the absence or presence of the next cognate nucleotide and as a result, PPi release and translocation will be slower or faster, respectively (95).

Ultimately, it is possible that Pol II nucleotide addition is described by two partially-rate limiting steps, bond formation and a conformational change of the trigger loop. Perhaps Pol II maintains a closed trigger loop conformation for longer, which would impede translocation and keep PPi from rapidly diffusing from the active center. Consequently, this would allow for the reverse reaction of nucleotide addition, pyrophosphorolysis, to occur. In support of this, the Lucius and Schneider groups found that Pol II pyrophosphorolysis was governed by a rate constant of ∼14 s^−1^ (75). Perhaps slow PPi release by Pol II, which allows for pyrophosphorolysis to occur, promotes careful selection of incoming NTPs which confers greater fidelity compared to Pols I and III—both of which contain *bona fide* ‘proofreading’ nuclease subunits. This feature of Pol II transcription may provide a way in which Pol II achieves high fidelity in absence of the association of a trans-acting proofreading factor, TFIIS. This is supported by *in vivo* fidelity data reported by the Lynch and Vermulst labs (96); Pol II is the least error-prone Pol.

In conclusion, multiple research groups support the notion that Pol II has two partially rate-limiting steps, one being bond formation. The second rate-limiting step remains to be revealed. Overall, there is good agreement in the rate of catalysis across research groups, while there is debate over Pol II’s NTP binding affinity. In comparison to Pol I, we find that Pol II is slower than Pol I and that PPi release may play a larger role in Pol II’s nucleotide addition mechanism compared to Pol I’s. Future investigations are needed to answer important outstanding questions, including what is Pol II’s binding affinity for each NTP? How do different NTPs influence the rate of bond formation, PPi release, and translocation? What characteristics of Pol II EC or the DNA environment influence the dynamics of the trigger loop? How do Pol II-specific TFs influence the mechanism of nucleotide addition?

## Pol III transcription elongation kinetics

### Pol III transcription elongation rate estimations *in vivo* and *in vitro*

Pol III transcription elongation is the least studied of the Pols. Pol III ECs can be monitored *in vivo* similar to Pol I. Using EM analysis of 5 S rDNA repeats in yeast, the Struhl lab estimated an elongation rate of ∼61 nt s^−1^ (72) (Table 3). As previously noted, their rate calculations relied on the assumption that each engaged Pol III EC would yield a 5 S rRNA transcript that was destined for ribosome formation. Substantial degradation of the 5 S rRNA would influence the elongation rate calculation.

**Table 3:** Pol III transcription elongation kinetic parameters

Pol III			
Parameter	Value	Method	Source
General transcription elongation estimations			
Elongation rate *in vivo*	∼61 nt s^−1^	EM analysis of 5 S rDNA	(72)
Elongation rate *in vitro*	∼(18–29) nt s^−1^	*SUP4* tRNA gene	(100)
	∼20 nt s^−1^		(101)
	∼57 nt s^−1^	Synthetic DNA template encoding four AMP and five GMP incorporations	(76)
Individual steps of nucleotide addition			
NTP binding	K_1/2_ = ∼100 μM ATP	Synthetic DNA template encoding one AMP incorporation	(76)
Bond formation	∼54 s^−1^	Synthetic DNA template encoding one AMP incorporation	(76)
Translocation		undetermined	

*In vivo* experiments were carried out at 30 °C and *in vitro* experiments were executed at (23–25) °C when noted by the authors.

The majority of *in vitro* Pol III transcription elongation research has monitored the appearance of end product RNA (97, 98, 99). Two groups using the same template—*SUP4* tRNA gene—have assigned average Pol III elongation rates that are in good agreement. Geiduschek and colleagues found that Pol III could oscillate between fast or slow kinetic paths over multiple nucleotide addition events (100). Specifically, Pol III synthesized RNA at heterogenous rates ranging from ∼(18–29) nt s^−1^ (100) (Table 3). Similarly, the Werner group measured an elongation rate of ∼20 nt s^−1^ (101) (Table 3). These values are smaller than what was estimated *in vivo*. Experimentally, both the Geiduschek and Werner groups incubated Pol III and DNA templates with TFIIIC and TFIIIB prior to providing NTPs. Since tRNA genes contain Type II, intergenic promoters, TFIIIC and TFIIIB bind over the gene. It is possible that the binding of TFIIIC and TFIIIB caused steric hindrance, ultimately slowing Pol III, or it is possible that Pol III synthesizes rRNA and tRNA at different rates. Or, as mentioned previously, the modest 1.7-fold difference between the *in vivo* and *in vitro* measurements may be a consequence of the differences between *in vitro* conditions and the nucleoplasm.

### Pol III nucleotide addition mechanisms *in vitro*

To determine the kinetic parameters that govern individual steps in the nucleotide addition cycle, the Schneider and Lucius groups monitored single AMP addition by Pol III *in vitro* (76). They globally fit their experimental data to a minimal kinetic model. They found that Pol III binds ATP with a K_1/2_ of ∼100 μM, and bond formation was governed by a rate constant of ∼54 s^−1^ (Table 3). To uncover the rate-limiting step of Pol III nucleotide addition, the reaction was perturbed by providing Sp-ATP-α-S instead of ATP, which led to a ∼40-fold decrease in the rate of bond formation (76). This suggests that AMP incorporation by Pol III, similar to Pols I and II, is at least partially rate-limited by bond formation.

Interestingly, the average rate of multi-nucleotide addition (four AMP and five GMP incorporations) was ∼57 nt s^−1^ (76) (Table 3). This is in good agreement with Pol III’s AMP bond formation rate constant, ∼54 s^−1^, and the *in vivo* EM estimate, ∼61 nt s^−1^. We conclude that Pol III *in vivo* and *in vitro* studies display good agreement but acknowledge that there are few studies available currently.

In conclusion, we find the rate of Pol III elongation is the most consistent in the literature compared to Pols I and II. Similar to Pols I and II, bond formation is a rate-limiting for Pol III. Dissimilar to Pol I, and similar to Pol II, Pol III nucleotide addition is less sensitive to sequence context. To advance our understanding of Pol III transcription elongation, many questions remain unanswered regarding its mechanism of PPi release and translocation. Further, how do nucleosomal *versus* bare DNA templates impact Pol III transcription elongation? Pol III initiates transcription from three unique promoters, how may the promoter type influence the rate of transcription elongation?

## Comparison of the Pols’ transcription elongation mechanisms

In summary, Pols I, II, and III share a similar binding affinity for ATP (Table 1, Table 2, Table 3). The binding affinities of Pols I, II, and III for each individual NTP have yet to be measured. While Pol II NTP binding has been investigated, the values are in disagreement across research groups (Table 2). There is a general trend in the rates of bond formation by the Pols: Pol I is the fastest, followed by Pol III, and Pol II is the slowest Pol. PPi release by Pols I, II, and III has yet to be directly monitored, while it has been in the bacterial transcription and DNA replication fields. Mechanistic work executed in bacteria (95, 102, 103, 104, 105, 106) reveal significant implications of fast or slow PPi release on the overall rate-limiting step of bacterial transcription. Thus, implementation of those PPi monitoring techniques (107) in the eukaryotic system is an important area of future research. Finally, most of what we know about translocation stems from Pol II research and those studies leave many questions open (Table 2). The mechanisms of translocation by Pols I and III have yet to be evaluated.

Collectively, these data show that there are both mechanistic differences and similarities across the Pols. Perhaps each biochemical difference was molded by distinct selective pressures stemming from their divergent cellular roles. It is of paramount importance to continue the investigation of Pol I, II, and III transcription elongation to advance our understanding of eukaryotic gene expression.

## Therapeutic implications of the divergence of the Pols

### Activities of Pols I, II, and III are essential for ribosome biogenesis

Pols I, II, and III are linked by the essential cellular process of ribosome biogenesis. Ribosomes are composed of two ribonucleoprotein subunits and are responsible for translating mRNA into polypeptide chains in all living organisms. The synthesis of ribosomes requires the activities of the three Pols. In yeast, Pol I synthesizes the 35 S rRNA, Pol III synthesizes the 5 S rRNA, and Pol II synthesizes mRNA encoding the 79 ribosomal proteins. The 40 S subunit is composed of the 18 S rRNA and 33 ribosomal proteins (108). The large 60 S subunit is composed of the 5 S rRNA, 5.8 S rRNA, 25 S rRNA, and 46 ribosomal proteins. Pre-ribosomes are assembled primarily in the nucleolus and exported to the cytoplasm to execute protein synthesis. In actively growing yeast, there are ∼20,000 ribosomes per cell, requiring 2000 ribosomes to be synthesized per minute (79), and they occupy 20% of the total volume of the yeast cytosol (109). Ultimately, ribosome biogenesis is an energetically costly process that requires the coordination of Pol I, II, and III transcription in all cells.

### Ribosome biogenesis as an anti-cancer target

Ribosome biogenesis is tightly linked to cell proliferation and, unsurprisingly, is highly dysregulated in cancerous cells (110). Interestingly, the link between ribosome biogenesis and cancer was first suggested over 125 years ago. In 1896, Pianese noted distinct, enlarged nucleoli in malignant cancer cells (111). Since then, many have observed nucleolar irregularities in cancer cells including increased size, increased number, and altered morphology (112, 113, 114, 115). Despite over a century of observations of nucleolar reorganization in cancer cells, only recently have researchers attempted to selectively target ribosome biogenesis as an anti-cancer strategy.

Attempts to target ribosome biogenesis fall into three general categories, transcription inhibitors, ribosome processing/assembly inhibitors, and translation inhibitors, reviewed previously (116, 117, 118, 119). Ultimately, most inhibitors are plagued by one core issue—adverse effects are not specific to cancer cells. While ongoing research is focused on delivering these potent anti-cancer drugs to cancer cells only (120), Pol I-targeting drugs may avoid this issue entirely.

### Rationale for chemotherapeutic targeting of Pol I

In the last decade, selective inhibition of Pol I has emerged as a viable anti-cancer strategy. The rationale for this strategy has been previously detailed (117, 121, 122). In brief, we know that ribosomal abundance correlates with cell growth rate. While all cells require translation, only hyperproliferative cells require excess ribosome production. Cancer cells need significantly more ribosomes than non-cancerous cells to promote tumor cell growth and proliferation. The first and rate-limiting step of ribosome biogenesis is Pol I transcription of the rDNA. Despite the essential role of Pol I in each eukaryotic cell, it is theorized that there is a window in which partial inhibition of Pol I would severely interfere with the rapid proliferation of cancer cells while non-lethally impacting the production of ribosomes in non-cancerous cells. It is hypothesized that non-cancerous cells have intact regulatory pathways to tolerate and overcome Pol I inhibition. In contrast, cancerous cells have “broken” these regulatory pathways (123) in order to upregulate ribosome biogenesis to its maximum. Therefore, they are more vulnerable to inhibition than non-cancerous cells.

These factors make Pol I a more attractive chemotherapeutic target compared to Pols II and III. Since Pols II and III synthesize non-ribosome related transcripts, targeting Pol II (*e.g.* CDK inhibitors, triptolide (124, 125)) or Pol III (*e.g.* ML-60218 (126, 127)) will inevitably impact other cellular processes and result in a higher risk of lethality. Similarly, the deployment of ribosome assembly inhibitors (*e.g.* Haemanthamine (128)) and translation inhibitors (*e.g.* rapamycin (129)) are challenging because all cells require protein synthesis. Taken together, the specific targeting of Pol I transcription may circumvent toxicity issues that plague other ribosome biogenesis inhibitors.

### Inhibitor BMH-21 exploits transcription elongation properties of Pol I

The two most studied Pol I inhibitors are CX-5461 (130) and BMH-21 (131). The Rice research group first described CX-5461 as a Pol I-specific transcription initiation inhibitor (130). They concluded that CX-5461 competes with SL1, the mammalian homolog of yeast CF, preventing SL1 from binding the rDNA promoter (130). Despite its anti-cancer effects (132, 133, 134, 135), later studies have shown that CX-5461 is not a Pol I-specific inhibitor (136, 137, 138, 139, 140). Rather, it acts through stabilizing G-quadruplex DNAs (136) and topoisomerase II poisoning (137), both resulting in DNA damage. Despite these liabilities, CX-5461 is advancing through Phase I clinical trials and has been shown to be generally well tolerated in patients with solid tumors (135).

BMH-21 is a Pol I-specific inhibitor that achieves its anti-cancer effects (141, 142, 143) without inducing DNA damage; Laiho and colleagues found that BMH-21 does not activate DNA damage signaling or repair pathways (*e.g.* ATM, ATR, H2AX, and KAP1) (131, 144). Instead, BMH-21 intercalates into GC-rich DNA (131, 145) which inhibits Pol I transcription and induces Pol I pausing (146, 147, 148). The Schneider research group found that the pausing was sequence specific; Pol I ECs preferentially paused directly upstream of G-rich DNA sequences (146). Ultimately, BMH-21 induces the degradation of Pol I (145, 147).

Since BMH-21 does not directly associate with Pol I ECs, how is BMH-21 a Pol I-specific inhibitor? There are at least two possible explanations, one could argue that BMH-21 preferentially intercalates the nucleosome-free, active rDNA repeats. We know that nucleosomes are a barrier to DNA intercalators (149). Therefore, it is possible that the unique chromatin environment of the rDNA makes it more susceptible to BMH-21 intercalation compared to the remaining nucleosome-bound genomic DNA transcribed by Pols II and III. Alternatively, it is possible that BMH-21 intercalates throughout the genome, but—due to the biophysical differences in transcription elongation by Pols I, II, and III, highlighted in the first half of this review—Pol I is the most sensitive to inhibition.

The Schneider, Lucius, and Laiho groups directly investigated the latter possibility by treating Pol I, II, and III ECs with BMH-21 *in vitro* and subsequently, measured its impact on their nucleotide incorporation mechanisms (148). If BMH-21’s selectivity was due to chromatin context, one would expect that Pol I, II, and III ECs treated with BMH-21 would be affected identically, since they utilized the same DNA template. In contrast, if Pol I is somehow uniquely sensitive to BMH-21, one would expect severe inhibitory effects of BMH-21 on Pol I, not Pols II and III. In fact, they observed the most severe inhibitory effect of BMH-21 on Pol I, with a lesser effect on Pol III, and no effect on Pol II (148). Pol I ECs treated with BMH-21, relative to the vehicle control, elongated slower and underwent paused states, ultimately forcing the enzyme into an alternative reaction scheme. The new scheme included off pathway steps from the linear scheme in which Pol I ECs undergo a reversible transition into a paused state. Pol III was slowed by BMH-21, but the mechanism of nucleotide addition was unchanged. Finally, Pol II was completely unaffected by BMH-21 treatment in comparison to the vehicle control. How can this be explained? Unique kinetic properties of Pol I render it vulnerable to inhibition by BMH-21 intercalation, whereas Pols II and III seem able to traverse these barriers more efficiently.

In comparison to Pols II and III, Pol I extends RNA the fastest (76); is the most sensitive to DNA sequence, evidenced by heterogenous nucleotide incorporation rate constants (87); and forms the least stable EC (76, 89). These unique Pol I properties could render Pol I especially vulnerable to the effects of BMH-21 treatment. This could be tested by treating a Pol I mutant lacking the A12 subunit (Pol I ΔA12) with BMH-21. In absence of A12, Appling *et al*. found that Pol I ΔA12 cannot cleave RNA efficiently, incorporates nucleotides ∼3-fold slower than WT Pol I, and forms a more stable EC (150, 151). If Pol I’s unique biophysical properties, in particular its fast rate of nucleotide addition and low EC stability, are responsible for sensitizing it to BMH-21 inhibition, we would expect Pol I ΔA12 to be largely unaffected by BMH-21 treatment. Lastly, potential chromatin effects may further sensitize Pol I ECs to BMH-21, but this idea remains to be tested.

## Conclusion

In conclusion, evolution has led to three mechanistically distinct eukaryotic Pols. Despite sharing the same essential steps in the nucleotide addition cycle, each Pol elongates RNA at different rates, substrate affinity, and via different mechanisms. This was revealed by many *in vivo* and *in vitro* studies. We argue that their kinetic differences are beneficial to the complex gene regulatory needs of higher organisms. Eukaryotes can deploy and modulate the activity of three unique molecular machines to specifically regulate the expression of subsets of genes. Ultimately, the divergent kinetic properties of Pols I, II, and III enable dynamic, precise gene control in eukaryotes.

The importance of understanding the divergent kinetic mechanisms of Pols I, II, and III extends beyond fundamental scientific benefit. On the translational side of Pol research, we cannot assess the efficacy of Pol I-targeting molecules without understanding their mechanisms in absence of treatment. As we advance our understanding of the mechanisms of Pol I, II, and III transcription, Pol I-specific differences/vulnerabilities will be revealed that can be targeted for chemotherapeutic effects. Taken together, mechanistic understanding of Pol I, II, and III transcription elongation fuel our insights into their evolutionary divergence and can be exploited in the development of novel Pol I-specific anti-cancer compounds.

## Conflict of interest

The authors declare no conflict of interest.
